# A Case of Malignant Disease

**Published:** 1857-08

**Authors:** N. O. Pearson

**Affiliations:** Palestine, Ill.


ARTICLE IV.—*A Case of Malignant Disease.* By N. O. PEARSON, M. D., of Palestine, Ill.

*History.*—Mr. Asa Jones, aged about 35, a gentleman of the dark bilious constitution; avocation that of a farmer; when convalescing from an attack of remittent fever (being still much debilitated), in feeding his horses, slipped and fell from the stable loft, resulting in an injury of the left inguinal region and testicle of the same side. Immediately after tumefaction of the testicle ensued, which swelled to the size of a goose-egg, accompanied with more or less fever at times, and producing great pain and tenderness of the part. Being confined to his bed for three months, the swelling gradually subsided, but did not entirely disappear, and would occasionally be troubled with pain of the injured part, although not very severe. Several years subsequently, the testicle began to enlarge again, accompanied at times with severe lancinating or burning pain, shooting up the course of the spermatic cord into the left lumber region, and down the sciatic nerve of the same side. These attacks of pain would come on at irregular intervals, and its severity often excruciating; particularly was this the case after a severe attack of pernicious fever, which occurred about eight years ago. Just subsequent to this period he lost the power of using with facility his inferior extremities, particularly the one corresponding with the injured testicle, and gained their use but slowly. For eight or ten months preceding his death (which took place in April, 1855), he was confined to his room, and a greater portion of his time to the bed, suffering extremely with pain, as I have mentioned. About three weeks prior to his death, erysipelas made its appearance upon the left foot, and extended nearly to the knee before its progress was arrested. I was called to see him by the request of the attending physician, E. J. French. He was found to present the following symptoms: there having been obstinate constipation for several days, which had resisted the action of all cathartic medicine; sickness of the stomach, regurgitation, everything taken into the stomach being rejected almost immediately; the action of the bowels themselves inverted, and feculent matter discharged from the mouth. Along with these symptoms were the following: a swelled testicle, painful and tympanitic condition of the whole abdomen, but more particularly the hypogastric and iliac regions; hiccough; great anxiety; cold sweats; a feeble and small pulse; general expression of prostration, and in fact symptoms simulating strangulated hernia, the testicle being enlarged to the size of a goose egg (as I have heretofore said), and presenting anteriorly a lobulated appearance, hard to the touch, with apparently considerable contusion (varicose condition of the superficial veins); the spermatic cord and inguinal glands were indurated, and integument covering the part corrugated, etc. Postmortem examination made six hours after death.

*Appearance and Condition of Organs.*—The tumor filling the left testicle cavity to its utmost capacity, extending and surrounding the spermatic cord, femoral artery, nerve and vein being connected to Poupart's ligament, and abdominal muscles, filling the whole iliac fossa, surrounding the iliac vessels, and attached to the inferior portion of the colon and rectum; the calibre of a considerable portion of the colon being much diminished (at least one third); it was also attached to the left and posterior portion of the bladder and rectum, extending up, and attached to the vertebral column as high as the diaphragm, but not connected to that organ, including in its mass the abdominal aorta and vena cava; these being much lessened, it also nearly surrounded the left kidney, the structure of which seemed healthy, though pressed somewhat out of shape. It was connected with the mesentary, and embraced several portions of the small intestines, between which were hardened and impacted fæces. Our attention was not directed to the pancreas (having so little time for the examination), but the presumption is, that it was included in the tumor, and its structure entirely changed; liver and spleen apparently healthy; lungs and heart not examined. The tumor would have weighed seven or eight pounds, and, by making incisions into it, fluid would escape freely, as if contained in sacks or cells, etc. The substance of some cells being soft, diffluent, like that of cream, and in others more vascular, presenting more of an intermixture of red streaks of blood. Not examined by the microscope.

